# Unique presentation of a giant mediastinal tumor as kyphosis: a case report

**DOI:** 10.1186/1752-1947-6-99

**Published:** 2012-04-04

**Authors:** Eleftherios D Spartalis, Theodore Karatzas, Petros Konofaos, Grigorios Karagkiouzis, Gregory Kouraklis, Periklis Tomos

**Affiliations:** 1 Second Department of Propedeutic Surgery, University of Athens, Medical School, "Laiko" General Hospital, Athens, Greece

## Abstract

**Introduction:**

Although posture distortion is a common problem in elderly patients, spinal deformity caused by a thymoma has not been previously reported. Thymomas are slowly growing tumors that predominantly cause respiratory symptoms.

**Case presentation:**

We report the case of an 83-year-old woman who was admitted with a giant mediastinal mass that had caused progressive spinal distortion and weight loss to our department. The clinical and laboratory investigations that followed revealed one of the largest thymomas ever reported in the medical literature, presenting as a mass lesion placed at the left hemithorax. She underwent complete surgical excision of the tumor via a median sternotomy. Two years after the operation, she showed significant improvement in her posture, no pulmonary discomfort, and a gain of 20 kg; she remains disease free based on radiographic investigations.

**Conclusions:**

In this case, a chronic asymmetric load on the spine resulted in an abnormal vertebral curvature deformity that presented as kyphosis.

## Introduction

Thymomas are rare neoplasms with a largely indolent growth pattern and various clinical symptoms [1]. The successful treatment of thymomas relies largely on complete surgical resection [2]. The first transsternal thymectomy was performed in 1939 by Alfred Blalock, and since then, a median sternotomy has been the standard surgical approach [2,3].

## Case Presentation

**Figure 1:**
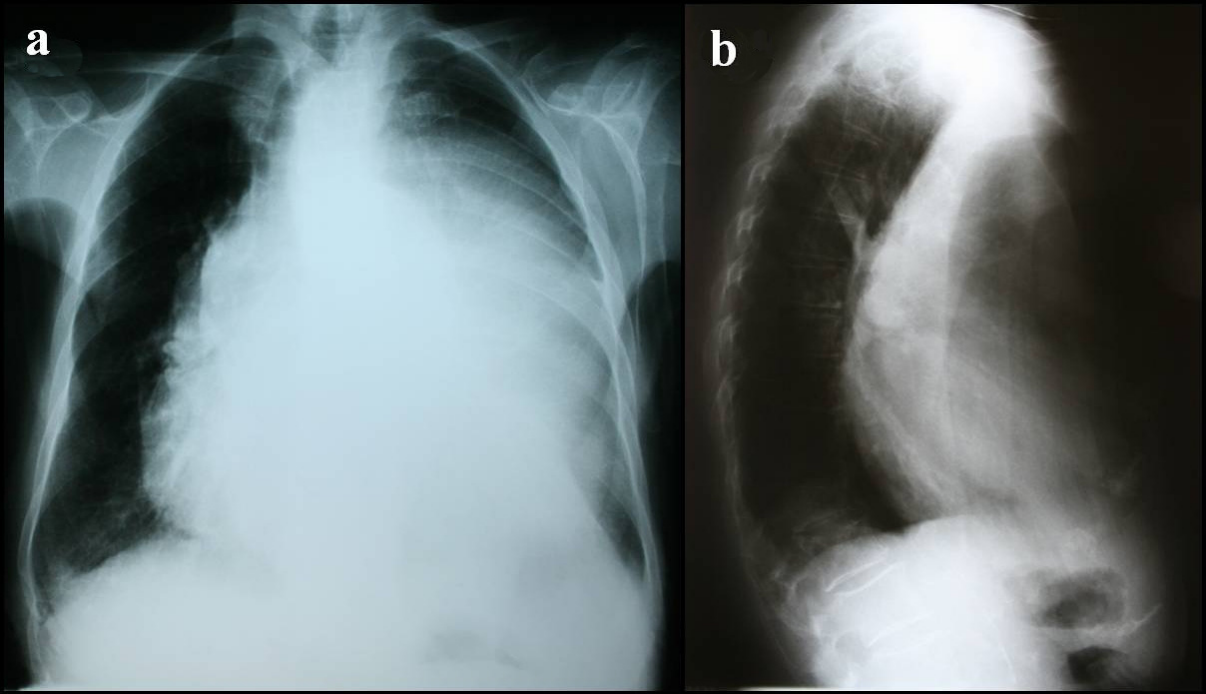
**Pre-operative posteroanterior chest radiograph and lateral chest radiograph**. Pre-operative (**a**) posteroanterior chest radiograph demonstrates a mediastinal mass that displaces the trachea and fills almost the entire left hemithorax. Lateral chest radiograph (**b) **shows mild kyphosis.

An 83-year-old Caucasian woman was admitted to the Department of Orthopedics with a chief complaint of progressive kyphosis, which had worsened over the previous six months. The patient complained of a progressive dysphagia and had lost approximately 20 kg over the previous six months. Posteroanterior and lateral chest radiographs (Figures 1a and 1b) demonstrated a mediastinal homogenous opacity partially filling the left hemithorax and causing tracheal displacement. She was then referred to our department for further investigation.

A physical examination revealed marked prominence of the left hemithorax. This region was dull to percussion and did not move with respiration. The intercostal spaces were bulging, and no breath sounds were heard on the left. The right hemithorax was clear and moved well with respiration. The remainder of the physical examination was unremarkable. Her general condition was good. Physical examination of the heart as well as an electrocardiogram (ECG) revealed no abnormal findings. Routine laboratory tests were within normal limits. Liver and renal function was very good.

**Figure 2:**
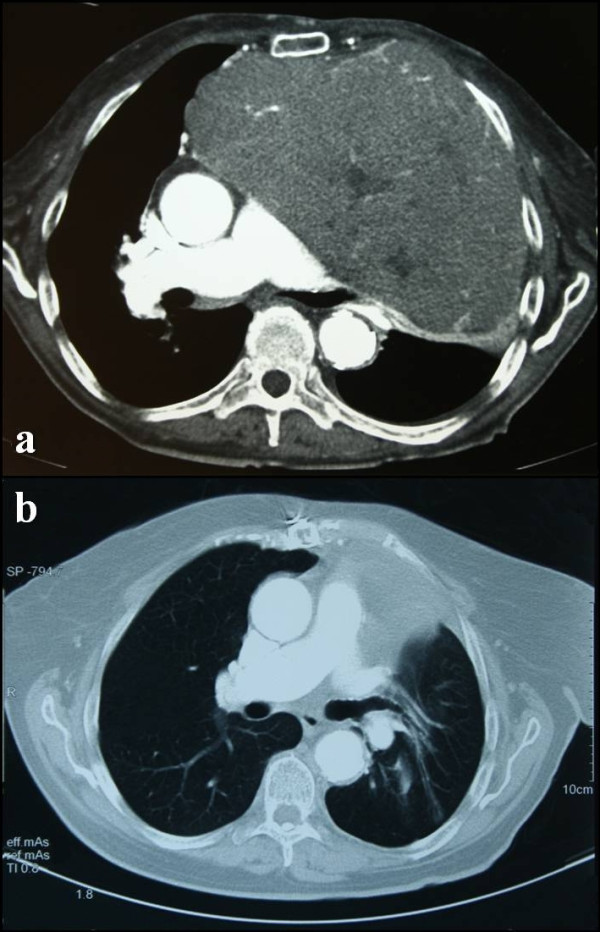
**Pre-operative and post-operative chest computed tomography scans**. (**a**) Pre-operative chest computed tomography scan reveals a soft-tissue-density lesion of the anterior mediastinum. (**b**) Post-operative chest computed tomography scan (same level imaging sections), performed two years after resection of the tumor, shows: no evidence of tumor, the esophagus returned to its anatomical position, decompression of the left main bronchus, expansion of the left lung, correction of the mediastinal width and regain of chest wall subcutaneous fat tissue.

The presence of a giant tumor was confirmed by computed tomography (CT) (Figure 2a) (size 2 × 20 × 11 cm). The CT scan showed that the tumor caused bilateral lung compression and tracheal and esophageal displacement. No fluid accumulation was seen. A CT-guided fine needle aspiration identified the mediastinal mass lesion as a possible thymoma.

**Figure 3:**
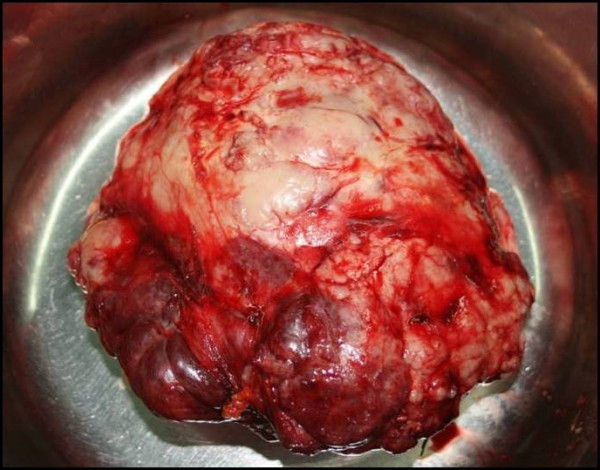
**Macroscopic image of the excised tumor**.

The patient subsequently underwent radical surgical excision of the tumor (Figure 3) via median sternotomy.

The total weight of the excised tumor was 2550 g (Table 1) [4-8]. Histological examination showed a thymoma [B1 according to the World Health Organization (WHO) classification, CD3(+), CD5(+), CD1(+), keratine(+), CD20(-) and CD30(-)]. The tumor was macroscopically and microscopically completely encapsulated (Masaoka stage I).

**Table 1:** Giant mediastinal thymomas in adults

Author	Year	Age (years)	Gender	Classification (WHO)	Tumor weight (g)
Sim	1992	83	Male	Type B1	n.d.
Selzner	1997	61	Female	Type A	1053
Agarwala	2003	65	Male	Type AB	1645
Gotte	2007	48	Female	Type A	1740
Yamazaki	2007	58	Female	Type A	n.d.
Santoprete	2007	73	Female	Type AB	n.d.
Moreno	2009	40	Male	Type AB	1725
Limmer	2009	52	Male	Type AB	1705

n.d., Not documented; WHO, World Health Organization

The patient was extubated in the operating room and then taken to the intensive care unit for two days of postoperative monitoring. The rest of her postoperative recovery was uneventful, and she was discharged on the tenth postoperative day.

Two years after the operation, the patient currently shows significant improvement of her posture, no pulmonary discomfort, and she has regained 20 kg of weight. Follow-up CT scans revealed no evidence of disease (Figure 2b). Her trachea and esophagus were returned to their anatomical location, the lungs were fully expanded and the mediastinum had returned to its normal width.

## Discussion

Thymomas typically present in the fourth or fifth decade of life and exhibit no gender predilection [1]. The presenting clinical symptoms in patients with thymomas are varied. When symptoms are present, they most often consist of cough, dyspnea, and other upper respiratory complaints [3]. In this case, the patient reported a chief complaint of spinal distortion associated with progressive symptoms of dysphagia and weight loss.

It is a commonplace observation that carrying a heavy load causes the carrier to lean forwards, and that in general, the heavier the load, the further forward the carrier leans [9]. The manner in which the body alters its normal alignment to achieve stability under conditions of load carriage has been examined thoroughly [10]. The body treats the load on the back as essentially a problem in balancing [11]. Thomas *et al. *found that if the vertical projection of the center of gravity is kept in a more or less constant position, it follows that lowering the position of a heavy weight on the back must cause the trunk to lean further forwards to keep the combined center of gravity of the body plus the weight in the same position [12]. Once an asymmetric load or degeneration occurs, the pathomorphology and pathomechanism in adult scoliosis is quite predictable. Asymmetric degeneration leads to increased asymmetric load and, therefore, to a progression of the degeneration and deformity [13]. In this case, the chronic, asymmetric load on the spine led to a progressive vertebral deformity that manifested as kyphosis.

Half of all thymomas present asymptomatically and are detected incidentally on radiographic imaging [1]. Imaging is an essential part of the workup, and in conjunction with the history and physical examination, it is often the only investigation needed prior to treatment [2]. Following the identification of a mediastinal mass on chest x-ray, a CT scan of the chest should be obtained. CT allows for the characterization of tumors and the assessment of possible invasion into surrounding structures [14]. CT studies with intravenous contrast administration are the preferred method for assessing vascular invasion and cystic components of the tumor [2]. Dynamic magnetic resonance imaging (MRI) has been examined as a potential way to improve the staging and differential diagnosis. Positron emission tomography (PET) has been examined as a method of tumor detection and for differentiating between invasive and non-invasive thymomas with mixed results [14].

The successful treatment of thymomas (both invasive and non-invasive) largely depends on complete surgical resection, if possible [15]. If an induction strategy for more advanced disease is being considered, or a diagnosis other than thymoma is likely, biopsy is recommended to confirm the diagnosis. Thymomas are staged according to their histopathological features (WHO-classification) as well as their clinical stage (Masaoka) [16].

Although thymomas are responsive to both chemotherapy and radiation, the mainstay of treatment is surgical resection [17]. Patients with inoperable tumors warrant a strategy of induction chemotherapy followed by a surgical reassessment post-therapy [18]. Despite the lack of prospective studies, adjuvant radiation therapy is generally recommended for any evidence of invasive disease, regardless of the degree of resection obtained. Since the ability to resect thymomas is closely associated with the tumor stage, the improvement of long-term prognosis for patients with advanced thymomas may ultimately depend upon developing effective multidisciplinary neoadjuvant treatment protocols that can downstage unresectable disease, allowing most patients to undergo a complete resection [15]. Lasting responses can be obtained both in the metastatic and recurrent setting, and novel therapies are currently being explored [17].

## Conclusions

Kyphosis in an adult with no previous history of any spinal curvature deformity should be considered as a possible symptom of an intrathoracic tumor. Imaging is an essential part of the investigation needed prior to surgical excision. CT-guided fine-needle aspiration biopsy is necessary for a definitive diagnosis and patient management. In the case of thymomas, complete surgical resection should be planned. Thymoma recurrence has been reported as late as 32 years after initial surgery [15]. As a result, patients with thymomas require life-long follow-up. The most important indicator of long-term prognosis is the completeness of resection [14]. Facing this case of a thymoma that presented as kyphosis, we achieved complete surgical excision of the tumor and improved the patient's posture and physical status.

## Consent

Written informed consent was obtained from the patient for publication of this case report and any accompanying images. A copy of the written consent is available for review by the Editor-in-Chief of this journal.

## Competing interests

The authors declare that they have no competing interests.

## Authors' contributions

ES analyzed and interpreted the patient's files, wrote the paper, assisted at the operation and looked after the patient postoperatively. TK and PT were the surgeons responsible for the patient, performed the operation and critically revised the manuscript. PK and GKa contributed to the writing. GKo is the supervising professor. All authors read and approved the final manuscript.
